# MULTISECTOR COLLABORATION DRIVES THE DEVELOPMENT OF THE FOURTH HEALTHY BRAIN INITIATIVE ROAD MAP

**DOI:** 10.1093/geroni/igad104.0853

**Published:** 2023-12-21

**Authors:** Kristen Clifford, James Appleby, Talyah Sands, Juan Rodriguez, John Shean

**Affiliations:** Alzheimer’s Association, Austin, Texas, United States; The Gerontological Society of America, Washington, District of Columbia, United States; Association of State and Territorial Health Officials, Arlington, Virginia, United States; Alzheimer’s Association, Chicago, Illinois, United States; Alzheimer’s Association, Chicago, Illinois, United States

## Abstract

Partnerships and collaboration across sectors played a critical role in the development of the fourth edition of the Healthy Brain Initiative Road Map (Road Map). The public health community, leaders in related fields, and people living with dementia contributed to the development and review. The Alzheimer’s Association and CDC invited 20 national leaders from public health, aging, academia, health care and health systems to guide the development of the fourth HBI Road Map as a part of the Leadership Committee. Their contributions included reviewing data, setting the overall direction of the review and leading five workgroups, composed of 68 subject-matter experts, who recommended actions on Risk Reduction of Cognitive Decline; Timely Detection, Diagnosis and Management of Cognitive Impairment; Dementia Caregiving; Health Equity; and Community Linkages. Health departments offered input through facilitated listening sessions and an open input period. Their feedback emphasized the importance increased information on health equity and partnerships in the Road Map. The Leadership Committee also advised continued partner engagement through the review process. This translated to review by the Alzheimer’s Association, CDC subject matter experts, external health equity experts, the Leadership Committee, workgroup members, the Healthy Brain Initiative Collaborative, the Alzheimer’s Association Early Stage Advisory Group and ultimately CDC leadership. This presentation will explore important themes that emerged throughout this process that shaped the design and evolution of the HBI Road Map series to its most recent iteration, as a guiding document with health equity and multi-sector collaboration action at its core.

